# DCCE-UNet: a difference and context-aware contrast enhanced framework for ultrasound image segmentation

**DOI:** 10.1186/s12880-025-01954-0

**Published:** 2025-11-04

**Authors:** Tiecheng Wang, Yehuan Xu, Linjie Hu, Huiling Liu, Kaidi Liu, Shuo Zhang, Hao Chen, Hua Guo, Shaobin Feng

**Affiliations:** 1 Department of Neurosurgery, Huantai County People’s Hospital, Huantai Avenue, Zibo, Shandong 256400 China; 2 Department of Neurology, Huantai County People’s Hospital, Huantai Avenue, Zibo, Shandong 256400 China; 3 Blood Purification Center, Huantai County People’s Hospital, Huantai Avenue, Zibo, Shandong 256400 China; 4 Department of Head and Neck Radiotherapy, Shandong Second Provincial General Hospital, Duanxing West Road, Jinan, Shandong 250021 China; 5 Department of Neurosurgery, Shandong Provincial Hospital Affiliated to Shandong First Medical University, Jingwuweiqi Road, Jinan, Shandong 250021 China

**Keywords:** Ultrasound image segmentation, Deep learning, Difference and context-aware contrast enhanced UNet

## Abstract

**Background:**

Accurate segmentation of lesion regions in ultrasound images (US) is critical for computer-aided diagnosis and clinical decision-making. However, the inherent challenges of US imaging—including blurred lesion boundaries, low tissue contrast, and irregular morphological variations—significantly hinder the reliability and generalizability of existing segmentation methods.

**Methods:**

To overcome these limitations, we propose Difference and Context-aware Contrast Enhanced UNet (DCCE-UNet), a novel structure-aware and context-enhanced segmentation framework that extends the U-Net backbone with three specialized modules: (1) the multi-source attention-guided semantic generation block, which improves multi-scale semantic extraction by adaptively aggregating complementary feature cues, (2) the bidirectional difference-aware attention module, which enhances boundary modeling by capturing directional feature differences, and (3) the context-aware guidance module, which integrates spatial contextual dependencies to refine feature representations. These components work in synergy to improve structural perception and spatial discrimination in US image segmentation.

**Results:**

Extensive experiments were conducted on two publicly available breast ultrasound datasets, BUSI and TN3K. The proposed DCCE-UNet consistently outperformed state-of-the-art models across key evaluation metrics, achieving a Dice coefficient of 79.85% and an IoU of 66.46% on BUSI, and reaching 89.29% Dice and 88.83% IoU on TN3K. Ablation studies further confirmed the effectiveness and complementary of each module in improving segmentation accuracy and boundary integrity, particularly in challenging cases involving small lesions, heterogeneous textures, and ambiguous contours.

**Conclusions:**

These results demonstrate that DCCE-UNet provides a robust and generalizable solution for automated ultrasound image segmentation. The proposed framework exhibits strong potential for real-world clinical deployment, with implications for improving diagnostic efficiency, reducing manual workload, and enabling reliable lesion analysis in ultrasound imaging workflows.

**Clinical trial number:**

Not applicable.

## Introduction

Medical ultrasound imaging has become a widely used screening modality in clinical practice due to its non-invasive, safe, and real-time characteristics. Its utility spans a wide range of applications, including routine screening, disease diagnosis, and interventional guidance across various anatomical regions such as the thyroid and breast. Accurate localization and delineation of pathological regions are critical for downstream tasks such as volumetric assessment, progression tracking, and therapeutic planning, wherein automated segmentation serves as a fundamental prerequisite for quantitative analysis and intelligent diagnostic systems [1].

However, ultrasound image segmentation remains highly challenging due to inherent imaging limitations. Unlike CT or MRI, ultrasound images often suffer from speckle noise, signal attenuation, and low contrast, which lead to indistinct boundaries, grayscale inhomogeneity, and irregular lesion morphology. These factors make it difficult to separate pathological regions from surrounding tissues, resulting in poor robustness and limited reliability in clinical decision-making:

To address these challenges, early ultrasound segmentation methods primarily relied on handcrafted features such as texture descriptors, edge maps, and region statistics. Techniques including gray-level co-occurrence matrices [2], wavelet transforms [3], and graph-based clustering [4, 5] were used to differentiate anatomical structures. Region-growing algorithms, local histograms, and morphological filtering were also employed to suppress speckle noise and enhance tissue boundaries [6]. Although these methods offered some interpretability and domain priors, their performance was highly sensitive to variations in image quality and lacked robustness under complex or noisy conditions, typical of real-world ultrasound applications.

The advent of deep learning has brought a new paradigm for ultrasound image segmentation, shifting from handcrafted feature pipelines to end-to-end representation learning. The introduction of fully convolutional networks [7] and U-Net [8] enabled end-to-end training and multiscale spatial representation, making them widely adopted baselines in medical image analysis. Subsequent work has proposed enhancements in feature encoding and spatial attention. For example, Squeeze-and-Excitation [9] and CBAM [10] reweight channel and spatial features to focus on informative regions, while multiscale architectures like DeepLabV3+ [11]. In addition, region-based integration-and-recalibration attention enables the network to focus on high contribution region representations and suppress less useful ones in optical coherence tomography images [12]. Recently, Vision Transformers [13] and hybrid CNN-Transformer models [14, 15] can capture long-range dependencies and global contextual relations, addressing limitations of convolutional locality. These models have demonstrated promising results in complex imaging scenarios, although their reliance on large datasets and computational overhead remains a practical concern. Beyond architectural improvements, attention has been paid to structure-aware and context-enhanced segmentation strategies. For instance, edge-guided modules [16], directional convolutions [17], and deformable attention mechanisms [18] aim to refine boundary localization and adapt to anatomical variability. In parallel, global-local context fusion [19], non-local modeling [20], and pyramid pixel context adaption  [21] have been employed to enhance spatial reasoning. Notably, contrastive learning-based segmentation [22, 23] has emerged as an effective approach to improve foreground-background separability by explicitly encoding semantic differences.

Despite these methodological advances, existing approaches still face limitations when applied to ultrasound images. In particular, they often fail to capture fine-grained structural differences between lesions and surrounding tissues, especially in regions with blurred boundaries or heterogeneous textures. Moreover, most models lack effective mechanisms to model direction-aware spatial dependencies and to enhance foreground-background separability, which are crucial for accurate lesion delineation. These limitations result in incomplete or over-smoothed segmentation masks, thereby limiting their translational potential in real-world ultrasound imaging workflows. We propose DCCE-UNet, a novel structure-aware segmentation framework that integrates three complementary modules into the classical U-Net backbone:

1. We propose DCCE-UNet, a novel framework that explicitly captures fine-grained structural variations between lesions and surrounding tissues, thereby alleviating the challenge of blurred boundaries and heterogeneous textures in ultrasound segmentation.
2. A multi-source attention-guided semantic generation block integrates multi-source features and guides attention learning with both spatial and contextual priors, enabling the network to more effectively delineate ambiguous lesion boundaries in ultrasound images.
3. A bidirectional difference-aware attention module effectively models anatomical orientation dependencies, which enhances the delineation of elongated or irregular structures that are often misrepresented by isotropic feature encoders.
4. Our context-aware guidance module enhances foreground-background separability through semantic contrast modeling, mitigating the loss of directional spatial context and recovering boundary-related information to achieve more accurate segmentation.
5. Experimental results on thyroid and breast lesions demonstrate that our method consistently outperforms several state-of-the-art baselines in terms of segmentation accuracy, highlighting its potential for real-world clinical application.

## Methods

**Fig. 1:**
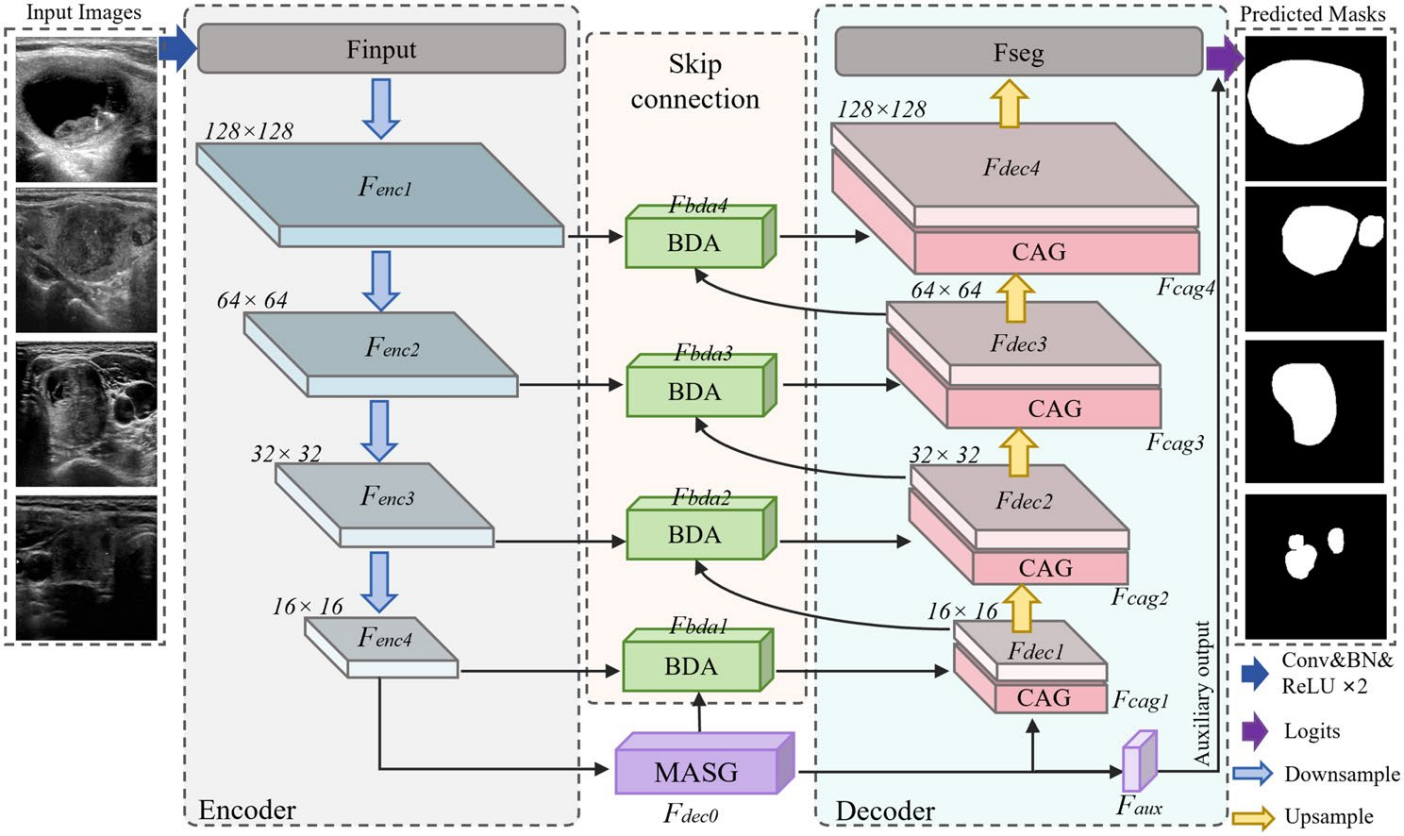
Our proposed DCCE-UNet architecture, which introduces a multi-source attention-guided semantic generation (MASG), bidirectional difference-aware attention (BDA), and context-aware guidance (CAG) into the standard U-Net framework. The overall design aims to enhance boundary detection, multi-scale feature representation, and spatial contextual modeling. These three modules progressively refine features by structure-aware and context-enhanced pathways to improve segmentation accuracy

We propose DCCE-UNet, a novel segmentation framework tailored for medical ultrasound imaging. The overall architecture is illustrated in Fig. 1. Based on the classical U-Net backbone, DCCE-UNet integrates structured and context-sensitive information into both encoder and decoder paths, in order to improve the model’s capability to capture structural boundaries and distinguish semantic regions. The input ultrasound image first passes through an initial double convolution module (Conv–BN–ReLU) to extract low-level features, which are subsequently downsampled by four encoding layers to form a hierarchical semantic representation. At the bottleneck of the encoder, we introduce Multi-source Attention-guided Semantic Generation (MASG) Block, which fuses multi-scale features and incorporates both spatial and variance-based attention mechanisms to enhance the model’s contextual understanding and semantic expressiveness, particularly in complex regions. During decoding, spatial resolution is progressively recovered through four upsampling layers. To enhance the effectiveness of skip connections and improve structural detail restoration, we embed two specialized modules in the decoder: the Bidirectional Difference-aware Attention (BDA) and the Context-Aware Guidance (CAG). BDA aligns features from the encoder and decoder while explicitly modeling their differences, thereby refining boundary localization and structural fidelity. In parallel, CAG employs directional depthwise separable convolutions and channel attention to capture orientation-sensitive spatial dependencies, enriching intra-region contextual representation.

### Multi-source attention-guided semantic generation

**Fig. 2:**
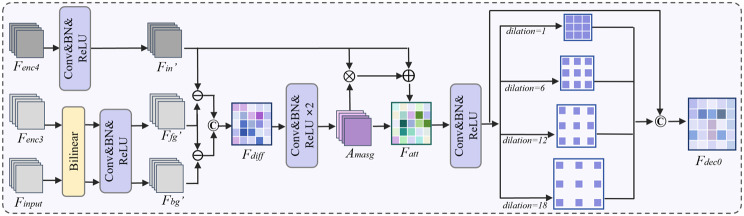
Overview of the proposed MASG block at the encoder bottleneck. The input bottleneck feature $F_{enc4}$ is contrasted with shallow foreground and deeper background references to generate an attention map $A_{\text{masg}}$. The resulting attention-enhanced feature $F_{att}$ is further enriched by four parallel dilated convolutions with dilation rates $d=\{1,6,12,18\}$, and concatenated to produce the final MASG output

To address the semantic ambiguity and contextual fragmentation commonly observed in ultrasound images, we design MASG at the encoder bottleneck, as shown in Fig. 2. The objective of this module is to strengthen semantic representation by integrating multi-source features and guiding attention learning with both spatial and contextual priors. The core idea is twofold: (i) capturing semantic contrast between foreground and background regions and (ii) aggregating multi-scale contextual dependencies via attention-based reweighting and dilated convolutions.

Let the input feature at the encoder bottleneck be denoted as $F_{\text{enc4}} \in \mathbb{R}^{B \times C \times H \times W}$. To provide semantic priors, we extract a shallow-level foreground reference $F_{\text{enc3}}$ and a deeper-level background reference $F_{\text{input}}$, both resized via bilinear interpolation to match $F_{\text{enc4}}$. After $1\times1$ convolution, batch normalization, and ReLU activation, we obtain the embedded features $F{^\prime}_{\text{in}}$, $F{^\prime}_{\text{fg}}$, and $F{^\prime}_{\text{bg}}$. The contrastive difference feature is then constructed as:

1 $$ F_{\text{diff}} = \text{Concat}(F{^\prime}_{\text{in}} - F{^\prime}_{\text{fg}},\; F{^\prime}_{\text{in}} - F{^\prime}_{\text{bg}})$$

$F_{\text{diff}}$ is then fed into a lightweight attention sub-network composed of two 1$\times$1 convolution layers to generate the attention map $A_{\text{masg}} \in \mathbb{R}^{B \times C \times H \times W}$. The final enhanced feature is obtained by residual fusion:

2 $$ F_{\text{att}} = F{^\prime}_{\text{in}} + A_{\text{masg}} \cdot F{^\prime}_{\text{in}}$$

Furthermore, to encode spatial context at multiple scales, we apply four parallel dilated convolutions to $F_{att}$ (with a kernel size of $3\times3$ and dilation rates $d=\{1,6,12,18\}$). Finally, $F_{att}$ is concatenated with the outputs of these dilated convolution branches to generate the final output of the MASG block:

3 $$\begin{aligned} F_{dec0} &= \text{Concat}\Big(F_{att}, \; \text{Concat}(\text{Conv}_{d=1}, \cr&\quad\; \text{Conv}_{d=6}, \; \text{Conv}_{d=12}, \; \text{Conv}_{d=18})(F_{att})\Big) \end{aligned}$$

To generate the auxiliary prediction, the decoder feature $F_{dec0}$ is refined by two convolutional layers and subsequently upsampled, resulting in the auxiliary segmentation output $F_{aux}$.

Through this design, MASG block encodes semantic contrast and contextual awareness, enabling the network to more effectively delineate ambiguous lesion boundaries in ultrasound images.

### Bidirectional difference-aware attention

For medical ultrasound image segmentation, skip connections of UNet are commonly used to transfer shallow encoder features to the decoder, facilitating the recovery of finer spatial information in downsampling. However, direct feature concatenation in skip connections often neglects the semantic and structural gaps between encoder and decoder stages, particularly in boundary regions where lesion and background textures are highly ambiguous. This mismatch may lead to blurred edges or feature redundancy. To address this, we design BDA to enhance skip connections by explicitly modeling cross-stage differences and refining boundary-sensitive features.

**Fig. 3:**
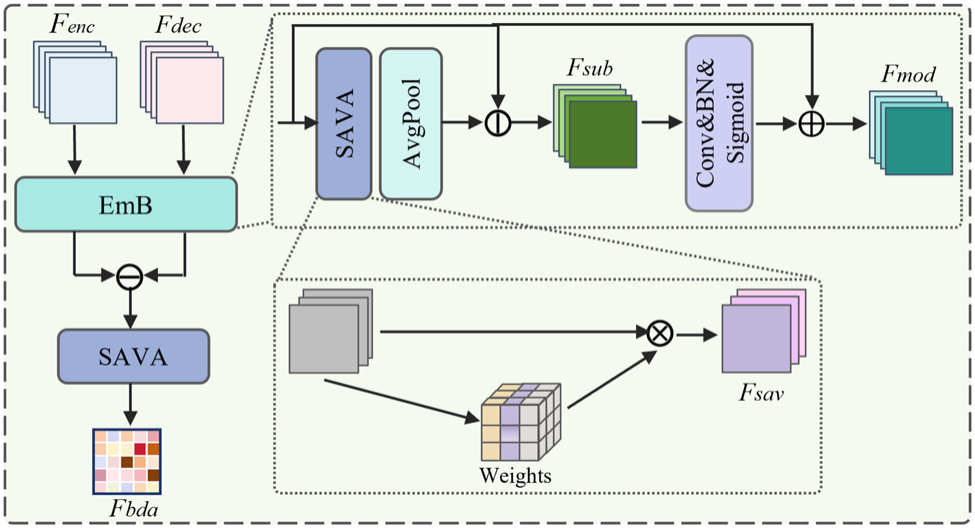
Overview of the proposed BDA (boundary difference attention) module. The encoder feature $F_{enc}$ and the upsampled decoder feature $F_{dec}$ are first embedded and compared to highlight boundary-sensitive differences. An attention weighting mechanism is then applied to refine $F_{enc}$, yielding the final BDA output $F_{bda}$

The BDA module improves the decoder’s ability to utilize encoder information along the skip pathways by integrating Embedding Block (EmB) and Spatial-Variance-aware Attention (SAVA) module. As shown in Fig. 3, BDA takes two inputs: a high-resolution encoder feature map $F_{enc} \in \mathbb{R}^{B \times C \times H \times W}$ and a low-level decoder feature $F_{dec}$ from the upsampling pathway, which are fed into the EmB module.

Within the EmB, SAVA is first applied to both $F_{enc}$ and $F_{dec}$ to perform spatial saliency modeling and statistical variance modeling. By computing channel-wise variance across spatial locations, SAVA highlights structurally informative regions and generates the feature $F_{sav}$, which guides the subsequent feature fusion process.

Then, the attention-enhanced feature $F_{sav}$ is subtracted from the original encoder feature $F_{enc}$ in a pixel-wise manner to obtain the fine-grained structural difference representation $F_{sub}$:

4 $$F_{sub} = F_{enc} - F_{sav}.$$

Next, $F_{sub}$ is fed into a lightweight channel attention module consisting of a $1 \times 1$ convolution, batch normalization, and a sigmoid activation. The resulting attention weights are applied through a residual weighting mechanism to enhance encoder representations, producing the fused output feature $F_{mod}$:

5 $$F_{mod} = F_{enc} + \sigma\big(BN(\text{Conv}(F_{sub}))\big) \cdot F_{enc}.$$

Here, $\sigma(BN(\text{Conv}(F_{sub}))) \cdot F_{enc}$ emphasizes regions with significant structural variations, while $F_{enc}$ preserves the original structure, achieving a balance between stability and discriminative enhancement.

Finally, the two intermediate results obtained by EmB are differenced again and passed through an additional SAVA module to further capture spatial variance information, yielding the final output of the BDA module, denoted as $F_{bda}$.

By combining feature embedding compression through the EmB, spatial-variance modeling via SAVA, and attention-guided difference fusion, BDA enhances the structural consistency of skip connections. Such design preserves boundary integrity and suppresses semantic noise, thereby strengthening the decoder’s capacity to reconstruct fine-grained lesion contours.

### Context-aware guidance

Boundary ambiguity is a major challenge in ultrasound image segmentation, as it often compromises spatial localization during the progressive refinement of decoder stages. To mitigate this issue, we introduce the CAG module, which enhances boundary sensitivity by explicitly modeling axis-aligned spatial dependencies. At each decoder stage, CAG adopts a dual-path design that separately encodes horizontal and vertical context to capture long-range correlations across rows and columns.

**Fig. 4:**
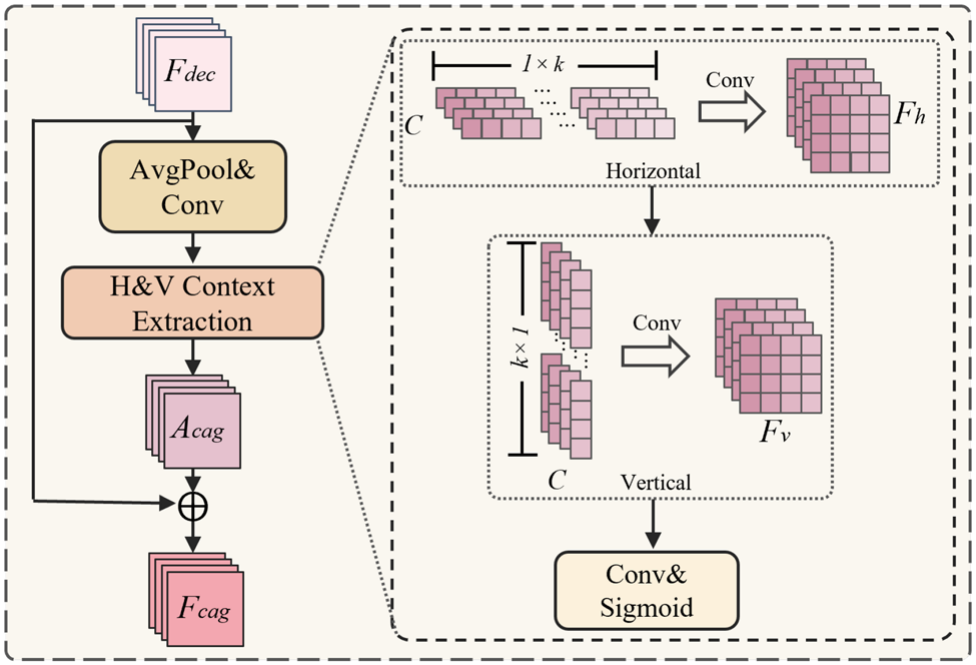
Overview of the proposed CAG module. It extracts horizontal and vertical contextual dependencies from $F_{dec}$ and generates the enhanced feature $F_{cag}$

As shown in Fig. 4, given a decoder feature $F_{dec}$, CAG first applies average pooling and a $1\times1$ convolution to embed contextual information. Then, the H&V Context Extraction branch separately encodes row-wise and column-wise dependencies through a horizontal $1\times k$ convolution to obtain $F_h$ and a vertical $k\times 1$ convolution to obtain $F_v$.

6 $$A_{cag} = \sigma \big(\text{Conv}(F_h + F_v)\big)$$

The outputs are combined and processed by a convolution and a sigmoid activation to generate the attention map, which modulates the original decoder feature. The final context-enhanced feature $F_{cag}$ is denoted as:

7 $$F_{cag} = F_{dec} + A_{cag} \cdot F_{dec}$$

$F_{cag}$ is then passed through two successive convolutional layers, yielding the final segmentation output denoted as $F_{seg}$.

Owing to its lightweight design and channel-independent formulation, the proposed CAG module introduces minimal computational overhead while effectively capturing axis-aligned spatial dependencies. By explicitly modeling the directional context, it improves the robustness of the decoder in ambiguous regions, improves structural consistency, and facilitates precise delineation of the lesion boundaries.

### Loss function

The loss function is designed to jointly optimize segmentation accuracy and boundary quality, which combines cross-entropy loss and Dice loss to balance per-pixel classification with region-level consistency. The overall training objective is defined as:

8 $$ \mathcal{L}_{\text{total}} = \mathcal{L}_{\text{ce}} + \mathcal{L}_{\text{dice}} + \lambda_{\text{aux}} \cdot \mathcal{L}_{\text{aux}}$$

Where $\lambda_{\text{aux}}$ denotes the weight balancing the auxiliary supervision loss $\mathcal{L}_{\text{aux}}$, defined as the cross-entropy loss between $F_{aux}$ and the ground truth (GT). $\mathcal{L}_{\text{ce}}$ and $\mathcal{L}_{\text{dice}}$ represent the cross-entropy loss and Dice loss, respectively, both computed between $F_{seg}$ and the GT.

## Results

### Implementation details and evaluation metric

**Datasets.** Experiments are conducted to verify the efficacy of our method based on two public datasets: TN3K and BUSI. (1) TN3K is a publicly available thyroid nodule ultrasound dataset comprising approximately 2,265 images [24]. We utilized a subset containing expert-annotated segmentation masks, including 1,527 benign and 738 malignant nodules. The segmentation labels are based on TI-RADS scoring and postoperative pathological confirmation. (2) BUSI includes 780 breast ultrasound images categorized into benign, malignant, and normal classes [25]. For this study, we selected 349 benign and 168 malignant images with available segmentation masks, discarding the normal category to focus on binary lesion segmentation. These datasets span multiple organ systems and various lesion types, providing a diverse and challenging benchmark for evaluating the effectiveness and generalization ability of segmentation models in different clinical scenarios.

**Implementation Details.** To ensure stable training and fair evaluation, all three datasets were randomly split into training, validation, and testing sets with a ratio of 8:1:1. During training, we used the Stochastic Gradient Descent (SGD) optimizer with a momentum of 0.9 and a weight decay of 5e-5. The initial learning rate was set to 0.01 and scheduled dynamically using the Cosine Annealing strategy. The total number of training epochs was set to 100, with a batch size of 8. All input images were resized to 224$\times$224 before being fed into the network. The loss function was a hybrid of cross-entropy loss, the Dice loss, and the weighted auxiliary supervision loss, designed to balance class-level discrimination and boundary sensitivity. To enhance reproducibility, a fixed random seed was used throughout the experiments. The model’s performance was continuously monitored on the validation set, and the parameters achieving the highest Dice score were saved for final evaluation on the test set.

**Evaluation Metrics**. To comprehensively evaluate the performance of our model on medical ultrasound image segmentation, we adopt four commonly used pixel-level evaluation metrics: Dice Coefficient, Intersection over Union (IoU), Pixel Accuracy, and Recall. These metrics reflect the model’s capability in foreground region detection and structural contour reconstruction from different perspectives. In addition, the comparison of Params and FLOPs further demonstrates the efficiency of our method.

### Experimental results

To comprehensively evaluate the effectiveness of our method, we conduct extensive comparisons on two publicly available ultrasound image segmentation benchmarks: BUSI and TN3K. The choice of baseline models for each dataset follows the conventions established in prior literature, ensuring fair and meaningful comparison consistent with existing research practices. Across both benchmarks, we also compare our model with the mainstream medical image segmentation networks that reflect diverse modeling strategies, including encoder-decoder architectures, attention mechanisms, feature fusion, and Transformer-based designs.

For the BUSI dataset, we compare DCCE-UNet with traditional encoder-decoder frameworks (e.g., *LAEDNet*, *FBCU-Net*), attention-based modules (e.g., *SECA-Net*, *SC-UNeXt*, *AOA-Net*), and advanced feature fusion strategies (e.g., *CFU-Net*, *DBUNet*). As shown in Table  1, our DCCE-UNet achieves superior performance across all metrics, including Dice, Accuracy, and IoU, with particularly notable improvements in Dice (79.85%) and IoU (66.46%), indicating its superiority in segmentation.

**Table 1:** Quantitative comparison of segmentation performance on the BUSI dataset

Method	Dice (%)	Accuracy (%)	IoU (%)	Recall (%)	Params (M)	FLOPs
*HEATNet* (2023) [1]	74.10	94.60	58.85	0.743	-	-
*LAEDNet* (2022) [26]	75.00	91.30	67.60	-	63.25	-
*GED-Net* (2024) [27]	64.79	96.10	69.40	80.10	55.92	30.16
*AOA-Net* (2025) [28]	67.50	95.70	59.90	62.10	43.78	26.35
*CFU-Net* (2023) [29]	78.57	96.38	66.26	76.50	-	17.93
*SC-UNeXt* (2024) [30]	75.29	**97.09**	63.66	66.12	7.89	8.67
*SECA-Net* (2024) [31]	77.53	96.71	65.81	72.00	-	-
*UMA-Net* (2025) [32]	74.30	95.70	59.10	67.73	36.30	5.13
*FBCU-Net* (2022) [33]	78.50	96.60	64.61	75.00	21.14	29.65
*EU*^*2*^*-Net* (2024) [34]	74.73	96.01	64.48	75.40	5.30	-
*DBUNet* (2025) [35]	79.11	95.73	65.44	81.28	0.88	20.84
*DCCE-UNet (Ours)*	**79.85**	96.82	**66.46**	**88.37**	8.80	8.70

Best results are highlighted in bold

Further evaluation is conducted on the TN3K dataset, where we compare against results reported in existing literature for methods such as *SHAN*, *TRFE+*, *BPAT-UNet*, *GSE-Net*, *ResUNet*, *DDTransUNet*, and *DRTNet*. The comparison also includes attention-based models (e.g., *GDSSSA-Net*, *MDSA-UNet*), Transformer-enhanced architectures (e.g., *HEATNet*, *DDTransUNet*), lightweight designs (e.g., *PEW-SegDiff*), and residual-enhanced networks. Our method consistently outperforms all baselines across Dice, Accuracy, and IoU, achieving a remarkable IoU score of 88.83%, which significantly surpasses the second-best result from *HEATNet* (81.06%). These results highlight the superior boundary modeling and region delineation capabilities of DCCE-UNet.

To ensure fairness, all comparative experiments adhere to the original data splits and *preprocessing* protocols reported in each method’s respective publication. For methods that are not publicly reproducible, we directly reference results reported in the literature. Under consistent experimental conditions, DCCE-UNet demonstrates superior performance on both datasets, confirming the robustness, *generalizability*, and effectiveness of our proposed approach in challenging ultrasound segmentation scenarios.

#### Comparative results

**Results on BUSI**. Table  1 displays the quantitative evaluation results of different segmentation methods on the BUSI dataset. It is observed that the proposed DCCE-UNet consistently outperforms all competing methods across three key evaluation metrics: Dice (79.85%), Accuracy (96.82%), and IoU (66.46%). Compared to the second-best method, *DBUNet* (Dice: 79.11%, IoU: 65.44%), DCCE-UNet achieves improvements of 0.74% in Dice and 1.02% in IoU, demonstrating its superior capability in region overlap accuracy and boundary delineation. Although *SC-UNeXt* achieves relatively high Accuracy (97.09%), its lower IoU (63.66%) indicates a limited ability to accurately segment small or irregular lesion regions. In contrast, DCCE-UNet not only maintains competitive overall accuracy but also achieves leading performance in both Dice and IoU, highlighting the effectiveness of its structure-aware segmentation design. Furthermore, the proposed method consistently outperforms several state-of-the-art models that incorporate attention mechanisms and multi-scale feature fusion strategies, including *SECA-Net*, *CFU-Net*, and *EU*$^2$*-Net*. DCCE-UNet achieves the highest Recall (88.37%), indicating its strong sensitivity to lesion regions. Moreover, the model maintains a competitive parameter size (8.80 M) and computational cost (8.70 GFLOPs), highlighting its efficiency and practicality in real-world clinical applications.

**Results on TN3K**. The quantitative evaluation results of different segmentation methods on the TN3K dataset are shown in Table  2. It can be observed that the proposed DCCE-UNet consistently achieves the best performance across all three evaluation metrics, achieving 89.29% on Dice, 97.34% on Accuracy, and 88.83% on IoU. Compared to *HEATNet*, DCCE-UNet brings substantial improvements of 3.79% in Dice and 7.77% in IoU, demonstrating its superior capability in accurate lesion delineation. Although some Transformer-based models, such as *HEATNet* and *DDTransUNet*, achieve competitive performance, they struggle to effectively capture complex lesion boundaries and model long-range spatial dependencies. It is worth noting that the TN3K dataset comprises more complex breast lesion ultrasound images with highly variable lesion shapes and blurred boundaries, which pose greater challenges for accurate segmentation. The consistently superior performance of DCCE-UNet on TN3K further highlights its effectiveness in handling complex and diverse ultrasound image segmentation scenarios. In addition, DCCE-UNet achieves the highest Recall (91.38%), indicating its strong sensitivity to lesion regions. Moreover, the model maintains a competitive parameter size (8.70 M) and computational cost (8.70 GFLOPs), highlighting its efficiency and practicality in real-world clinical applications. These results further emphasize the robustness and applicability of our approach in segmenting complex breast ultrasound images.

**Table 2:** Quantitative comparison of segmentation performance on the TN3K dataset

Method	Dice (%)	Accuracy (%)	IoU (%)	Recall (%)	Params (M)	FLOPs
*SHAN* (2022) [36]	84.61	96.73	73.59	79.80	39.28	34.88
*TRFE+* (2023) [37]	83.30	97.04	71.38	73.91	25.45	33.82
*BPAT-UNet* (2023) [38]	83.64	97.22	71.87	85.57	-	15.96
*MDSA-UNet* (2025) [39]	80.20	97.05	70.39	-	6.65	4.54
*GSE-Net* (2025) [40]	81.97	97.34	72.24	-	8.84	1.88
*PEW-SegDiff* (2025) [41]	78.64	91.94	74.29	78.81	-	-
*GDSSA-Net* (2025) [42]	84.27	96.25	73.03	81.63	8.75	-
*ResUNet* (2025) [43]	83.77	97.18	75.09	89.14	18.30	26.07
*HEATNet* (2023) [1]	85.50	97.00	81.06	85.90	-	-
*DDTransUNet* (2024) [44]	82.31	96.94	73.82	84.80	15.50	31.00
*DRTNet* (2025) [45]	84.96	95.74	73.82	80.32	16.36	10.19
*DCCE-UNet (Ours)*	**89.29**	**97.34**	**88.83**	**91.38**	8.80	8.70

Best results are highlighted in bold

#### Impact of loss design

**Fig. 5:**
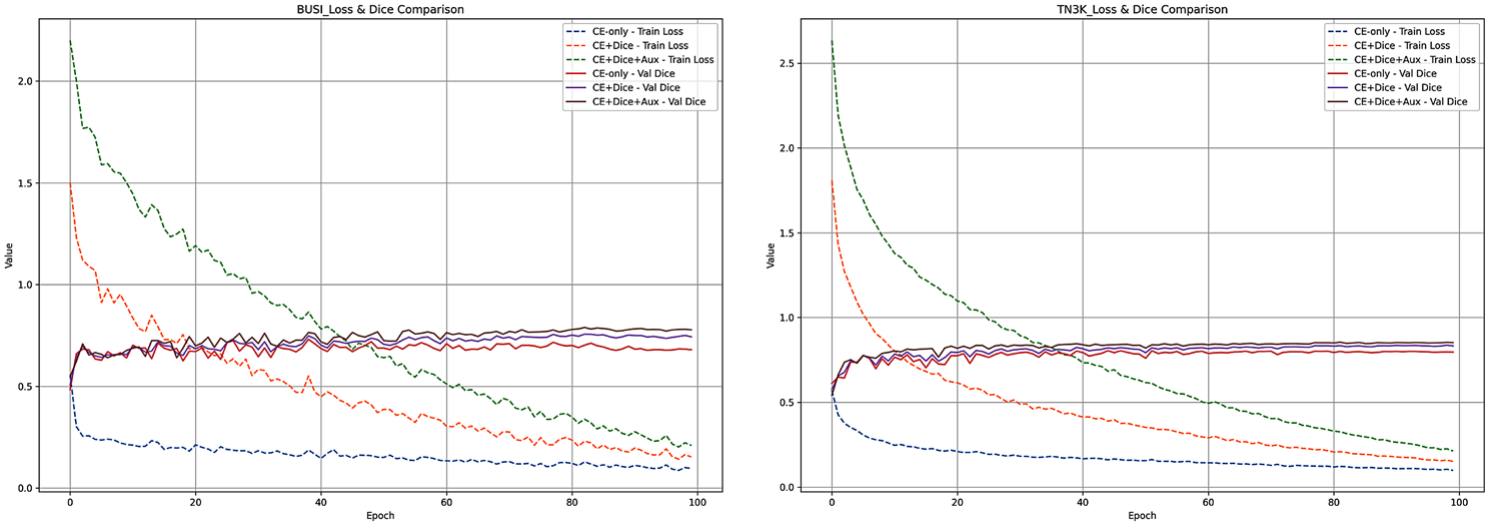
Training and validation curves of DCCE-UNet on the BUSI and TN3K datasets under different loss configurations. The combination of Cross-Entropy, Dice, and auxiliary supervision yields faster convergence and higher Dice scores compared to other configurations

Figure 5 presents the training curves of our method on the BUSI and TN3K datasets under different loss configurations. The dashed lines denote the training loss, while the solid lines represent the Dice coefficient on the validation set. As shown, the “CE-only” configuration exhibits a slower decline in training loss and relatively low Dice performance, suggesting limited ability to capture fine-grained details and boundaries. Incorporating Dice loss (CE + Dice) accelerates the decrease in training loss and substantially improves validation Dice, highlighting its role in enhancing boundary detection and region-level consistency. Further adding auxiliary supervision leads to the most favorable training dynamics, with faster convergence, reduced overfitting, and the highest Dice score, demonstrating its effectiveness in improving gradient flow and model generalization. Overall, these results confirm that the joint use of Cross-Entropy loss, Dice loss, and auxiliary supervision is critical for achieving robust convergence, precise boundary delineation, and reliable segmentation performance on ultrasound images.

#### Ablation study

To validate the effectiveness of each proposed module, we conducted ablation experiments on both the BUSI and TN3K datasets.

Ablation Results on BUSI: As shown in Table  3, the baseline model implemented as a standard U-Net architecture achieves 74.46% Dice, 59.69% IoU, and 95.84% Accuracy. Incorporating the Bidirectional Difference-aware Attention Module (BDA) achieves improvements across all key metrics, with the IoU improving to 61.30%, indicating enhanced boundary modeling and spatial discrimination. Further incorporating the CAG results in a Dice score of 77.59% and a Precision of 69.24%, demonstrating its effectiveness in promoting channel-wise feature refinement and reducing false positive predictions. In addition, the ablation results also include efficiency-related metrics. Compared to the baseline UNet, the proposed modules slightly increase the number of parameters and FLOPs, but achieve a significant reduction in FPS as the network becomes more complex. Nevertheless, the trade-off remains acceptable considering the accuracy improvements. Finally, integrating the Multi-source Attention-guided Semantic Generation Block (MASG) yields the best overall performance, achieving 79.85% Dice, 66.46% IoU, and 72.83% Precision. It indicates that MASG can capture multi-scale semantic information and effectively distinguish lesion regions, thereby enhancing robustness in segmenting small lesions and handling ambiguous boundaries.

**Table 3:** Ablation study results on the BUSI dataset

Model	Dice	IoU	Acc	Rec	Spe	Pre	Params	FLOPs	FPS
UNet	74.46	59.69	95.84	86.45	96.56	65.85	4.30	7.76	441.55
+BDA	76.01	61.30	96.07	87.17	96.76	67.38	4.49	8.19	381.78
+BDA+CAG	77.59	63.39	96.36	88.24	96.99	69.24	6.77	8.54	320.62
+BDA+CAG+MASG	**79.85**	**66.46**	**96.82**	**88.37**	**97.47**	**72.83**	**8.80**	**8.70**	**212.46**

Ablation Results on TN3K: The ablation results on the TN3K dataset are shown in Table  4. The baseline U-Net obtains a relatively low performance with 79.77% Dice and 66.34% IoU. The incorporation of BDA leads to a substantial improvement, increasing Dice to 85.41% and Sensitivity to 93.92%, indicating its sensitivity to lesion regions. Further incorporating CAG improves Dice and IoU to 88.01% and 78.59%, respectively. When all modules are integrated into DCCE-UNet, the model achieves the best performance on TN3K, with 89.29% Dice, 80.65% IoU, and 87.28% Precision, consistently outperforming other configurations across all metrics. In addition, Table  4 also reports the model complexity (*Params* and *GFLOPs*) and efficiency (*FPS*). The results show that while adding modules slightly increases the number of parameters and computational cost, the full DCCE-UNet still maintains a reasonable complexity (8.80 M Params, 8.70 GFLOPs) and achieves a practical inference speed of 209 FPS, which is sufficient for real-time clinical applications. This indicates that the proposed BDA, CAG, and MASG modules each contribute different performance gains by enhancing boundary modeling, channel attention, and multi-scale feature extraction, respectively.

**Table 4:** Ablation study results on the TN3K dataset

Model	Dice	IoU	Acc	Sen	Spe	Pre	Params	FLOPs	FPS
UNet	79.77	66.34	94.93	82.47	96.65	77.24	4.30	7.71	426.15
+BDA	85.41	74.54	96.11	93.92	96.41	78.31	4.49	8.19	373.32
+BDA+CAG	88.01	78.59	96.95	92.26	97.60	84.14	6.77	8.54	320.62
+BDA+CAG+MASG	**89.29**	**80.65**	**97.34**	**91.38**	**98.16**	**87.28**	**8.80**	**8.70**	**209.45**

**Fig. 6:**
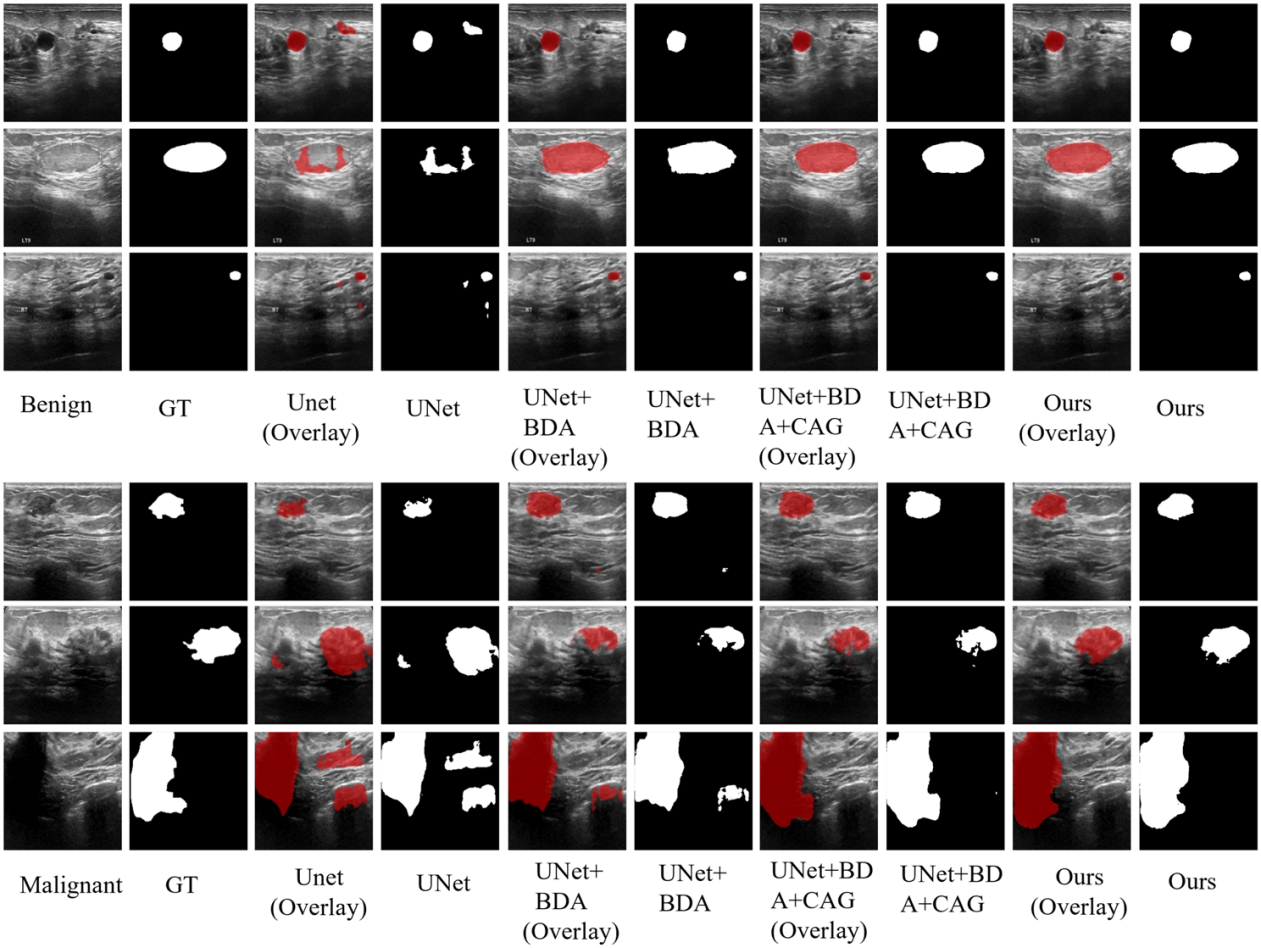
Qualitative comparison illustrates the visual superiority of the proposed modules in improving lesion localization and boundary delineation on the BUSI dataset, where DCCE-UNet produces segmentation results that are more consistent with the ground truth (red)

**Fig. 7:**
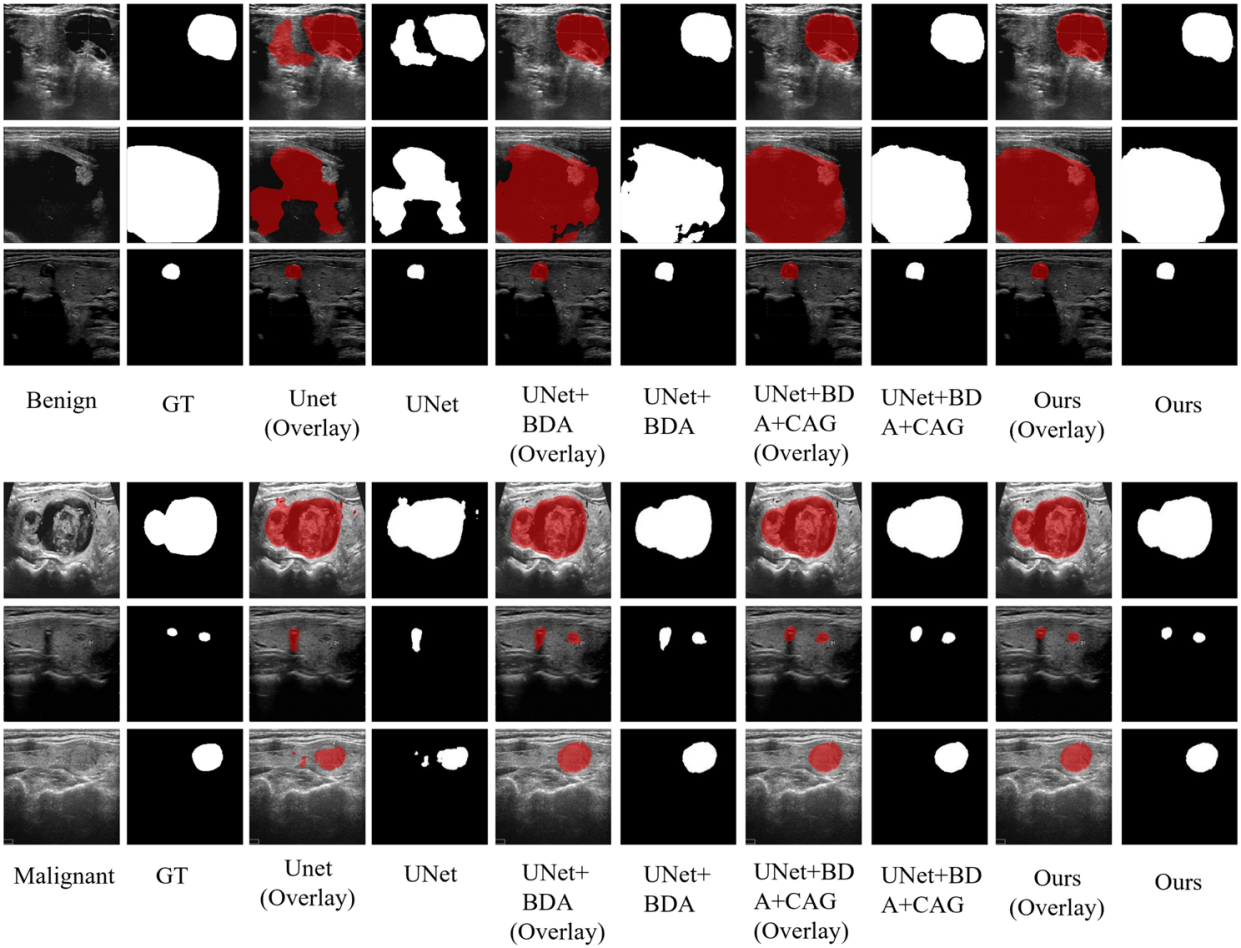
Our DCCE-UNet illustrates accurate segmentation on TN3K across challenging cases, including small lesions and blurred boundaries, with precise area overlapping and boundary alignment

The qualitative segmentation results in Figs. 6 and 7 also demonstrate the effectiveness of each proposed module of DCCE-UNet. It is observed that the baseline U-Net exhibits inaccurate segmentation results, particularly in cases with low contrast or blurred structures. The incorporation of BDA leads to better alignment with lesion boundaries and improved structural completeness. The subsequent addition of CAG further enhances boundary discrimination and reduces over-segmentation in complex regions. The final DCCE-UNet generates accurate, smooth, and complete lesion contours that closely match the ground truth across all cases, demonstrating its superiority in local and spatial modeling for both lesion localization and boundary delineation.

## Discussion

This study presents DCCE-UNet, a novel structure-aware and context-enhanced segmentation framework designed to address the challenges of ultrasound image segmentation. By the integration of three complementary modules (MASG, BDA, and CAG), the proposed model demonstrates significant improvements in segmenting lesions with complex morphology, blurred boundaries, and low contrast.

Our experimental results on thyroid and breast ultrasound datasets indicate that DCCE-UNet outperforms several state-of-the-art methods in multiple quantitative metrics, including Dice, IoU, and accuracy. In particular, one of the major challenges in ultrasound segmentation is the presence of blurred and indistinct lesion boundaries. DCCE-UNet explicitly addresses this issue through its modular design: the MASG module enhances semantic contrast to improve foreground–background separability, thereby sharpening indistinct lesion margins; the BDA module effectively captures subtle structural variations between encoder and decoder stages, aiding in precise contour localization; the CAG module strengthens the model’s ability to capture long-range anatomical dependencies along directional orientations, which helps to recover boundary continuity. Together, these components contribute not only to improved boundary delineation under challenging conditions but also to the model’s robust generalization ability across datasets with varying image quality and lesion types.

Despite the strong performance, several limitations merit further discussion. First, while DCCE-UNet is lightweight compared to Transformer-heavy architectures, it still introduces additional parameters relative to plain U-Net, which may affect real-time deployment in edge devices or resource-constrained clinical settings. Future work could address this by exploring model compression techniques (e.g., pruning and quantization) and knowledge distillation to reduce computational overhead while retaining accuracy. Second, although the model demonstrates generalization across multiple datasets, its performance under extreme imaging artifacts (e.g., shadowing, reverberation) has not been fully explored. Third, the model currently focuses on binary or single-class lesion segmentation; extending it to multi-class, multi-instance scenarios would further enhance its clinical utility. Moreover, while the present framework is designed for 2D static ultrasound images, many clinical tasks (e.g., echocardiography) involve video sequences with spatiotemporal information. Extending DCCE-UNet with temporal modeling modules (e.g., ConvLSTMs, 3D CNNs, or video transformers) represents a promising future direction to capture motion dynamics and broaden its applicability to real-world ultrasound video analysis.

Future research will also explore the integration of self-supervised pretraining to alleviate annotation burdens, and the incorporation of multi-modal ultrasound information, such as elastography or Doppler flow, to provide richer contextual cues. Finally, embedding the proposed model into a complete diagnostic pipeline, for instance, coupling segmentation with lesion classification or malignancy prediction, can further enhance its application in real-world ultrasound-aided diagnosis workflows.

## Conclusion

This paper presents a novel structure-aware and context-enhanced segmentation framework, DCCE-UNet, to achieve accurate and robust lesion segmentation in ultrasound images. Our DCCE-UNet integrates three complementary modules into the standard U-Net architecture: MASG for improving multi-scale semantic representation, BDA for enhancing boundary modeling, and CAG for capturing direction-aware spatial dependencies. The MASG further strengthens the model’s ability to extract discriminative features across different scales, ensuring accurate segmentation even in challenging cases with blurred boundaries, small lesions, or heterogeneous textures. The BDA explicitly models fine-grained structural differences to better distinguish lesion boundaries, while the CAG enriches intra-region contextual encoding through the integration of horizontal and vertical spatial information. Through the progressive refinement of spatial and semantic features, DCCE-UNet enhances both regional localization and boundary delineation, contributing to more reliable and precise lesion segmentation. Extensive experiments on two public ultrasound image datasets, BUSI and TN3K, demonstrate the effectiveness and robustness of the proposed method, consistently outperforming state-of-the-art approaches in both quantitative metrics and qualitative assessments. The proposed DCCE-UNet shows promising potential for clinical applications in ultrasound-based computer-aided diagnosis and interventional planning, offering a reliable tool for accurate lesion assessment and supporting real-world medical decision-making.

## Data Availability

The datasets used in this study include the TN3K dataset, which is publicly available at https://github.com/haifangong/TRFE-Net-for-thyroid-nodule-segmentation, and the Breast Ultrasound Images (BUSI) dataset, available at https://scholar.cu.edu.eg/?q=afahmy/pages/dataset.
